# Motion of Air Bubbles in a Cement Slurry

**DOI:** 10.3390/ma16196433

**Published:** 2023-09-27

**Authors:** N’dri Arthur Konan, Eilis Rosenbaum, Mehrdad Massoudi

**Affiliations:** 1 National Energy Technology Laboratory, 3610 Collins Ferry Road, Morgantown, WV 26507, USA; ndri.konan@netl.doe.gov; 2 NETL Support Contractor, 3610 Collins Ferry Road, Morgantown, WV 26507, USA; 3 National Energy Technology Laboratory, 626 Cochran Mill Road, Pittsburgh, PA 15236, USA; eilis.rosenbaum@netl.doe.gov

**Keywords:** yield stress fluid, cement slurry, gas migration, Herschel–Bulkley fluid, Bingham fluid, VOF, bubble

## Abstract

The dynamics of air (gas) bubbles in a column of cement slurry is examined numerically. The air injected at the bottom of a laboratory-scale column through a porous distributor plate spatially distributes and migrates as a swarm of bubbles throughout the slurry toward the freeboard. The two-phase system of the cement slurry and the air bubbles is modeled using the conservation equations of mass and linear momentum in the framework of the volume-of-fluid (VOF) approach. The cement slurry is modeled using the Herschel–Bulkley and Bingham fluid models. Results show that the mean Sauter diameter and the mean rise velocity of the bubbles decrease with the gas flow rate. Meanwhile, it is found that the rising of the bubbles is controlled by breakup events, along with relatively weak path instabilities of the bubbles resulting in relatively straight trajectories, independent of the gas flow rate. The extent of the yielded region appears larger for the Herschel–Bulkley model compared to the Bingham fluid model (by approximately 10%).

## 1. Introduction

Environmental risks associated with the integrity of wellbores remain an active concern for oil and gas industries (see, e.g., Vidic et al., 2013 [1]). Survey-based analyses of thousands of reports of conventional and unconventional oil/gas wells in Pennsylvania/USA by Ingraffea et al. (2014) [2] suggest that these integrity issues may be correlated to cementing operation failures (e.g., cement density, premature gelation, high permeability in the cement slurry, etc.). It is known that cemented Aeolian sand-fly ash backfill material can be used to fill the mining area to help with the damage caused by underground coal mining (Yang et al., 2023 [3]). Bonett and Pafitis (1996) [4] discuss circumstances that may impair the cementing of the wellbores on the basis of three states of the cement: (1) a slurry that is a granular-type fluid with an evolving yield stress that further exhibits a vulnerability to the invasion of the gas from the surrounding formation because of pressure differentials between these two media as the cement hardens; (2) a fully self-supporting two-phase material marked by continued setting and accelerated hydration that result in an internal shrinkage, a weak solid structure, subject to viscoelastic fractures caused by the influx of gas; and (3) an elastic and brittle material where gas migration can no longer occur, although gas flows through interfacial channels can take place. These authors further discuss four gas migration patterns (i.e., bubbling, slug, interface, and rising plume) through the cement slurry with a dependence on the gas flow rate (typically within the range of 10^−9^ to 10^−7^ m^3^/s). Wellbore cementing challenges are many, and great technical efforts are put forward to prevent compromising the integrity of wellbore cementing (see, e.g., Renteria et al., 2022 [5]; Maleki and Frigaard, 2019 [6]). It is known that air bubbles can have detrimental effect on concrete and cement, reducing the compressive strength of hardened concrete during the later stages of cement hydration process. For a review of important issues related to gas migration in cement, see Tao et al. (2021) [7].

Several studies point toward the design of cements with controlled and enhanced properties that could prevent the migration (see, e.g., Velayati et al., 2015 [8]; Ahmed et al., 2020 [9]; Moradi and Nikolaev, 2016 [10]; Teodoriu and Asamba, 2015 [11]). Velayati et al. [8] use additives in the cement slurries of a laboratory scale and report significant reductions in the migration. However, following Bonett and Pafitis (1996) [4], one would expect a bubbling migration pattern for the lower flow rates reported by Velayati et al. (2015) [8]. Given that the cement slurry could still show vulnerabilities despite improved cement designs, additional insights into the process of gas migration should help better understand the strength of the wellbore and improve and anticipate mitigations. The following questions need to be considered. (1) How does the migrating gas spatially distribute throughout the slurry column? (2) What are the size and aspect ratio distributions of the gas bubbles? (3) When the bubbles are not motionless, how fast do they rise in the slurry?

Cement slurries, in general, behave as complex nonlinear fluids that may exhibit viscoelasticity, thixotropy, yield stress, shear-thinning effects, etc. (see the review by Tao et al., 2020 [12]; Banfil, 2006 [13], 1993 [14]; and Rodrigues and de Souza Mendes, 2019 [15]). As indicated by Yuan, et al. (2022) [16], the thixotropy of cement becomes crucial when a cement slurry is used for mine-out area backfilling, where it should be pumped easily but should also become a solid-like material at very small velocities. An extensive literature discussing fundamental aspects of air bubble injection, detachment, growth, and rising mechanisms in different types of non-Newtonian fluids exists. These studies mostly have focused on single or a few bubbles with controlled injection points.

Ghosh and Ulbrecht (1989) [17] examine experimentally the formation and detachment of a single bubble in stagnant shear-thinning and viscoelastic fluids characterized by the power-law and the two-parameter Oldroyd models, respectively. For the sake of comparative analysis, they also look at a viscous Newtonian fluid. Gosh and Ulbrecht [17] observe a delayed detachment and slower growth rate of the bubbles injected in the viscoelastic fluid compared to both the shear-thinning and the viscous Newtonian fluids. They also report that while the bubble volumes are found to increase with the air flow rate, higher bubble volumes are obtained with fluids of higher apparent viscosities. They observe that bubbles elongate in the direction of their vertical axis at higher gas flow rates for Newtonian and low-viscosity fluids, whereas for the viscoelastic fluid, elongation phenomenon occurs even at low gas flow rates, and bubble elongation increases with the gas flow rate. Within a column filled with yield stress Herschel–Bulkley fluid, Terasaka and Tsuge (2001) [18] similarly investigate experimentally the formation and the growth of single gas bubbles injected through a nozzle. The authors report a strong correlation between the rheological properties of the fluids and the bubble volumes. Indeed, their observations show that, at a fixed gas injection flow rate, the larger the yield stress is, the larger the bubble volume.

Dubash and Frigaard (2004) [19] study the permanent entrapment and moving conditions of single air bubbles in a viscoplastic fluid (modeled as a Herschel–Bulkley fluid). They derived two stopping conditions by relying on variational-based minimizations of the symmetric part of the velocity gradient and the stress fields, respectively. These conditions, which can be identified as realizability criteria, stipulate that above a critical yield stress (or a critical Bingham number), the bubble will not rise in the fluid. Dubash and Frigaard [19] show that these stopping conditions are independent of the fluid constitutive parameters, namely, the consistency index and the power-law exponent; they explain that until a bubble begins to move, it only sees the yield stress of the fluid. Indeed, the bubble rises when the force caused by the buoyancy of the bubble is sufficient to overcome the yield stress. In a follow-up experimental study, Dubash and Frigaard (2007) [20] discuss the validity of those conditions against measurements of the bubble rise velocities by assuming that bubbles have axisymmetric shapes for the sake of simplicity. The experiments consist of injecting single bubbles of several different sizes through a nozzle, where the column is filled with a fluid assumed to behave as a Herschel–Bulkley fluid. The experiment shows that the yield stress parameters are beyond the range of the stopping conditions previously derived by Dubash and Frigaard (2004) [19]. They also show from an energy-based analysis that the bubble buoyancy is balanced by both the yield and the surface tension stresses; they report that the surface tension effects appear to be significant and more important for smaller bubbles. Dubash and Frigaard [20] also observe a rapid increase in the bubble terminal velocity as the bubble-to-column radius ratio increases, while above a certain bubble-to-column radius ratio they report a maximum terminal velocity.

Stein and Buggisch (2000) [21] investigate the possibility of initially static small bubbles to rise when subjected to pulsations, i.e., an additional mechanism beyond the buoyancy allowing the bubbles to overcome the yield stress. Their experiments show that there exists a pulsating amplitude threshold with a dependence on the bubble size below which the bubble does not rise. In addition, they observe that larger bubbles rise faster than small ones for a given pulsation amplitude. Similarly, Iwata et al. (2008) [22] investigated experimentally the enhancement of a single bubble rising velocity by controlling the fluid pressure through a sine-wave-based mechanical vibration. Their measurements are for a shear-thinning Carreau–Yasuda fluid (i.e., not a yield stress fluid) and show that higher oscillation frequency results in higher shear rates, along with a substantial decrease in the fluid effective viscosity around the bubble. The authors observe that the bubble’s rising velocity increases by two orders of magnitude, which they partly attribute to the reduction in the apparent viscosity in the vicinity of the bubble. In a recent work, De Corato et al. (2019) [23] investigated numerically Iwata et al.’s experiments and reported that the bubble’s radial motion reduces the viscosity of the surrounding fluid and subsequently may greatly reduce the friction coefficient of the bubble. This mechanism can be used to control the enhancement of the bubble’s rising velocity.

For the flow of interest in this work (which could also be applicable to geothermal applications), the gas is mostly expected to invade the cement slurry through the interface between the external periphery of the wellbore annulus of the stagnant cement slurry and the surrounding porous medium formation. This implies that the gas is injected over a surface, and therefore, multiple bubbles can be expected to detach (either at once or successively). In addition, the pressure fluctuations along with the change in the influx rate of the gas percolating the porous formation could generate flow pulsations at any of the gas invasion locations. Such a flow is understood to be adequately approximated by the investigation of a column of cement slurry subject to a continuous air (gas) injection at the bottom through a porous distributor plate.

In this paper, we look at the motion of bubbles as they rise in a cement slurry column, where the cement is modeled as a Herschel–Bulkley fluid. Quantities such as the geometrical dimensions of the bubbles, along with their rise angle, position, and velocities, are discussed as the flow rate is varied. To gain further insight and to check our formulation and numerical scheme, we simulate the experiment of Terasaka and Tsuge (2001) [18] on the formation and growth of a single bubble in a yield stress fluid, an aqueous solution of xanthan gum. In the next section, the conservation equations of the mass and the linear momentum within the framework of the volume-of-fluid (VOF) approach is presented. An overview of the numerical methods and the simulation conditions are followed by thorough discussions of the results. Finally, some concluding remarks are drawn, and possible future works are discussed.

## 2. Mathematical Framework

Cement is a multicomponent complex material with microstructure, undergoing chemical and physical changes, and its constitutive modeling can be a daunting task. A cement slurry is composed of particles, water, air, etc. The presence of bubbles in the cement slurry adds further complexity to the problem at hand. The mixture, as a suspension when flowing, behaves as a nonlinear fluid with shear-thinning effect, thixotropy, yield stress, etc. In the paste form, cement can behave as a visco-plastic or visco-elastic material, and after it has hardened, it can behave as a nonlinear poro-elastic material. The most common rheological models used are the Bingham model and the Herschel–Bulkley model (Konan et al., 2022 [24]; Plancher et al., 2022 [25]). To mathematically model a cement slurry, in general, we can use (1) a suspension rheology approach where a single constitutive relation is provided for the Cauchy stress tensor T (see Tao, et al., 2020 [12]), or (2) use a two-phase (multi-phase) approach where constitutive relations are needed for the two stress tensors $T_{1}$ and $T_{2}$ for cement particles and water, respectively (with $\phi_{1}$ and $\phi_{2}$ as the volume fractions of the two phases), and an interaction force $f_{I}$ (see Massoudi, 2003 [26]), or (3) use a modified suspension approach where a stress tensor T for the suspension and a flux vector N are required for the whole system (see Tao et al., 2019 [27]). The volume-of-fluid (VOF) approach is similar to the modified suspension approach, and it presents a computationally feasible method for certain multiphase applications. In this paper, we use this method.

### 2.1. Volume-of-Fluid (VOF) Method

Here, we provide a brief description of the traditional VOF method, and later, we generalize this to the case of a cement slurry with air bubbles. Let us consider the physical domain, Ω, of two immiscible fluids (i.e., the cement slurry and the gas). Following Hirt and Nichols (1981) [28] and Nichols et al. (1980) [29], let us introduce a volume fraction, $\phi \left(x,t\right),$ whose value of unity at any point (x∈Ω) indicates the full presence of a given fluid (i.e., cement slurry) and zero otherwise. A value of the volume fraction ϕ between 0 and 1 signifies the presence of an interface between the two fluids. At any point x∈Ω in time $t\left(>0\right)$, the two-fluid system is considered as “one-fluid” with a total velocity $u\left(x,t\right)$ and a density $\rho \left(x,t\right)$. This “one-fluid” is assumed to be incompressible:

(1) div u=0

where “div” stands for the divergence operator. The density of the “one-fluid” is defined using the volume fraction $\phi \left(x,t\right),$ such that:

(2) $$\rho \left(x,t\right)=\phi \rho_{1}+\left(1-\phi \right)\rho_{2}$$

where $\rho_{1}$ and $\rho_{2}$ are the constant densities of the fluids labeled 1 and 2, i.e., the cement slurry and the gas, respectively.

The linear momentum of the “one-fluid” is assumed to be described by:

(3) $$\frac{\partial }{\partial t}\left(\rho u\right)+div\left(\rho uu\right)=divT+\rho b+⟦\Sigma ⟧$$

where T is the Cauchy stress tensor, b is the body force vector, and $⟦\Sigma ⟧$ is the source term because of the interfacial interaction between the two fluids; this accounts for the stress jump at the interface and the immiscibility assumption.

The stress for the traditional “one-fluid” VOF approach is described through a single pressure (p) and a single viscosity (μ) by relying upon the volume fraction, such that:

(4) $$\mu \left(x,t\right)=\phi \mu_{1}+\left(1-\phi \right)\mu_{2}$$

where $\mu_{1}$ and $\mu_{2}$ are the viscosities of the fluids labelled 1 and 2, respectively. At any point x∈Ω in time $t\left(>0\right)$, we assume that the Cauchy stress tensor for the “one-fluid” is given by:

(5) $$T\left(x,t\right)=-p\left(x,t\right)I+\mu \left(x,t\right)A_{1}$$

where I is the identity tensor, and $A_{1}=grad\;u+{\left(grad\;u\right)}^{T}$ is the kinematical tensor, related to D, the symmetric part of the velocity gradient [$A_{1}=2D$].

The volume fraction ϕ, which serves as a flag identifying the presence of a given fluid, is governed by a convection–diffusion type equation of the “one-fluid”, such that:

(6) $$\frac{\partial \phi }{\partial t}+u\cdot \nabla \phi =0\;$$

where ∇ is the gradient operator.

### 2.2. Constitutive Relations

#### 2.2.1. Interfacial Interaction between the Two Fluids

The difference in the physical properties such as density or viscosity of the two immiscible fluids induce interfacial interactions. The net tensile force acting on the interface of curvature κ, separating the two fluids can be modeled as (Brackbill et al., 1992 [30]):

(7) $$⟦\Sigma ⟧=\int_{V\left(t\right)}\sigma \kappa \left(x\right)n\left(x\right)\delta \left[n\left(x_{s}\right)\cdot \left(x-x_{s}\right)\right]dx$$

(8) $$⟦\Sigma ⟧≅\sigma \kappa \left(x_{s}\right)n\left(x_{s}\right)$$

where σ is the surface tension, n is the unit normal vector to the interface, and δ is the Dirac function.

The curvature κ of a surface at a point $x_{s}$, along the unit vector $\hat{n}$ to the interface (within a given cell volume), are assumed to be given by:

(9) $$\kappa =-div\;\hat{n}$$

(10) $$\hat{n}=\frac{\nabla \phi }{\left|\nabla \phi \right|}$$

where ∇ is the gradient operator. Brackbill et al. (1992) [30] argue, based on practical computational challenges, that the normal vector in n (Equation (8)) can be replaced by −∇ϕ, so that the source term because of the interfacial interaction between the two fluids is given by:

(11) $$⟦\Sigma ⟧=\sigma \left[div\left(\frac{\nabla \phi }{\left|\nabla \phi \right|}\right)\right]\nabla \phi$$

This interaction term can appear in two-phase flows, as one of the many interaction forces, in the form of:

$$⟦\Sigma ⟧=\omega \nabla \phi$$

along with other forces such as drag, lift, etc. (see Massoudi 2003 [26]).

#### 2.2.2. Stress Tensor

For our problem, we assume that air (fluid 2) is injected into cement slurry (fluid 1). The densities $\rho_{1}$ and $\rho_{2}$ of the cement slurry and the air (gas), respectively, are considered constant, as indicated by Equation (2).

In general, as shown by Tao et. al. (2019 [27], 2020 [12]), a cement slurry may exhibit yield stress, thixotropy, shear-dependent viscosity, concentration-dependent viscosity, etc. In this paper, we assume that the cement slurry can be modeled as a Herschel–Bulkley fluid, and we ignore thixotropy and concentration-dependent viscosity. Thus, we assume that the stress in the “one-fluid” of the VOF method is given by:

(12) $$T\left(x,t\right)=-p\left(x,t\right)I+\tau \left(x,t\right)=-p\left(x,t\right)I+\tau_{1}\left(x,t\right)+\tau_{2}\left(x,t\right)$$

where $\tau_{1}$ and $\tau_{2}$ are the stress tensors associated with the cement slurry and the air, respectively.

For the cement slurry, we assume it behaves as a Herschel–Bulkley fluid, where $\tau_{1}$ (in its proper tensorial form as given in Macosko, 1994 [31]) is given by:

(13) $$\tau_{1}\left(x,t\right)=\phi \left[k{\left|{II}_{2D}\right|}^{\frac{n-1}{2}}+\frac{\tau_{0}}{{\left|{II}_{2D}\right|}^{1/2}}\right]\left(2D\right)\;\;\;for\;{II}_{\tau_{1}}^{1/2}>\tau_{0}$$

(14) $$D=0\;\;for\;{II}_{\tau_{1}}^{1/2}\leq \tau_{0}$$

where $\tau_{0}$ is the yield stress, k is the consistency index, and n is the power-law exponent, a measure of the nonlinearity of the fluid, related to the shear-thinning effects (when n<0) or shear-thickening effects (when n>0). ${II}_{\tau_{1}}$ and ${II}_{2D}$ are the second invariants of the stress tensor $\tau_{1}$ and of the symmetric part of the velocity gradient tensor $2D=\left(grad\;u\right)+{\left(grad\;u\right)}^{T}$, respectively. Note that the Bingham fluid is obtained from the above Equation (13) when the power-law exponent n=1, i.e., the viscosity is now independent of the shear rate.

The stress tensor $\tau_{2}$ for the air (gas) is given by:

(15) $$\tau_{2}\left(x,t\right)=\left(1-\phi \right)\mu_{2}\left(2D\right)$$

where $\mu_{2}$ is the viscosity of the air. For the sake of simplicity, the flow is assumed to be laminar. We should note here that in the VOF method, as opposed to the general case of two-phase flow formulation, we only have one velocity u and only one velocity gradient.

## 3. Numerical Approach

The computational fluid dynamics (CFD) finite volume method from the open-source toolbox/library, OpenFOAM, is used on unstructured grids to solve the mass and the linear momentum conservation equations, using the solver “*multiphaseInterFoam*” customized with non-Newtonian viscosity libraries. To avoid the numerical implementation challenges associated with the discontinuity (singularity) in the stress tensor field $\tau_{1}$ of the cement slurry fluid between the unyielded and the yielded regions, the regularization method is used, and the stress tensor of the cement slurry $\tau_{1}$ is replaced with an ϵ-dependent small parameter, such that:

(16) $$\tau_{1,\epsilon }\left(x,t\right)=\phi \mu_{1,\epsilon }\left({\left|{II}_{2D}\right|}^{1/2}\right)\left(2D\right)$$

where the ϵ-dependent viscosity $\mu_{1,\epsilon }$ is approximated according to Papanastasiou (1987) [32] by:

(17) $$\mu_{1,\epsilon }\left({\left|{II}_{2D}\right|}^{1/2}\right)=k{\left|{II}_{2D}\right|}^{\left(n-1\right)/2}+\frac{\tau_{0}}{{\left|{II}_{2D}\right|}^{1/2}}\left[1-exp\left(-\frac{{\left|{II}_{2D}\right|}^{1/2}}{\epsilon }\right)\right]$$

This approach has recently been used by Konan et al. (2022) [24] in their study of a cement slurry, as well as by Espinoza et al. (2022) [33] in a similar context of the Herschel–Bulkley fluid coupled with the VOF approach to investigate the primary cementing processes of an oil well. From a numerical standpoint, the advection of the volume fraction ϕ through (Equation (6)) may undesirably lead to the smearing of ϕ and, thus, losing the sharp interface separating the two fluids. To preserve this discontinuity nature of the volume fraction with the transition region as narrow as possible, the so-called “interface compression” method is introduced through an additional convective term in the advection equation of ϕ (see, e.g., Rusche, 2003 [34]; Berberović et al., 2009 [35]; Hoang et al., 2013 [36]; Cifani et al., 2016 [37]). Indeed, the advection of the volume fraction in each fluid, advected by $u_{1}$ and $u_{2}$, are:

(18) $$\frac{\partial \phi }{\partial t}+div\left(\phi u_{1}\right)=0\;$$

(19) $$\frac{\partial }{\partial t}\left(1-\phi \right)+div\left(\left(1-\phi \right)u_{2}\right)=0\;$$

Adding Equations (18) and (19), we have:

(20) $$div\left(\phi u_{1}+\left(1-\phi \right)u_{2}\right)=divu=0\;$$

where the total velocity defined as $u=\phi u_{1}+\left(1-\phi \right)u_{2}$ would result in $u=u_{1}$, when the interface is strictly sharp. Introducing a relative velocity $u_{r}$ (to “compress” the interface) as:

(21) $$u_{r}=u_{1}-u_{2}\;$$

we have:

(22) $$\phi u_{1}=\phi u+\phi \left(1-\phi \right)u_{r}\;$$

Substituting Equation (22) in Equation (18), we obtain:

(23) $$\frac{\partial \phi }{\partial t}+div\left(\phi u\right)+div\left(\phi \left(1-\phi \right)u_{r}\right)=0\;$$

where the additional convective term is only active within the narrow interface region restricted by $\phi \left(1-\phi \right)$. This convective term aims at avoiding the use of a special discretization scheme for the convection in the advection equation of ϕ, which could allow a better resolution for sharper interfaces. The relative (or the interface compression) velocity is assumed to be given by:

(24) $$u_{r}=c_{\phi }\left|u\right|n\;$$

where $c_{\phi }$ is an adjustable compression factor, and n is the unit normal vector to the interface. 

Using Equation (10) for the unit vector, the relative velocity becomes:

(25) $$u_{r}=c_{\phi }\left|u\right|\frac{\nabla \phi }{\left|\nabla \phi \right|}\;$$

Now, substituting Equations (4)–(25) in Equation (3), along with the incompressibility condition of the “one-fluid”, the basic equations that need to be solved numerically are:

(26) div u=0

(27) $$\frac{\partial }{\partial t}\left(\rho u\right)+div\left(\rho uu\right)=-\nabla p+div\left\{\left[\phi \mu_{1,\epsilon }\left({\left|{II}_{2D}\right|}^{1/2}\right)+\left(1-\phi \right)\mu_{2}\right]\left(2D\right)\right\}\;+\rho g+\sigma \left[div\left(\frac{\nabla \phi }{\left|\nabla \phi \right|}\right)\right]\nabla \phi$$

(28) $$\rho \left(x,t\right)=\phi \rho_{1}+\left(1-\phi \right)\rho_{2}$$

(29) $$\frac{\partial \phi }{\partial t}+u\cdot \nabla \phi +div\left[c_{\phi }\left|u\right|\phi \left(1-\phi \right)\frac{\nabla \phi }{\left|\nabla \phi \right|}\right]=0\;$$

In the context of the experiments of gas migration into a column of cement slurry as described in the experimental section of this paper, the boundary conditions can be formulated as follows.

At the distributor plate:

(30) $$u=U_{g}$$

(31) n·∇p=0

where $U_{g}$ is the velocity of the gas injected at the distributor plate.

At the wall:

(32) u=0

(33) n·∇p=0

At the outlet of the column:

(34) n·∇u=0

(35) $$p=p_{out}$$

where $p_{out}$ is the outlet pressure, set as the atmospheric pressure.

Initially, the column of the slurry is at rest; thus:

(36) $$u\left(x,t\right)=0$$

(37) $$p\left(x,t\right)=0$$

## 4. Parameters Used in the Simulation

The slurry was prepared using the preconditioned class H cement (Headrick et al., 2023 [38]). The rheological properties from the fitting of the data to the Herschel–Bulkley and the Bingham models are summarized in Table 1. The density of the slurry is 1970 kg/m^3^. The surface tension is not measured; however, for the sake of simulations, σ = 0.07 N/m is used. 

**Figure 1:**
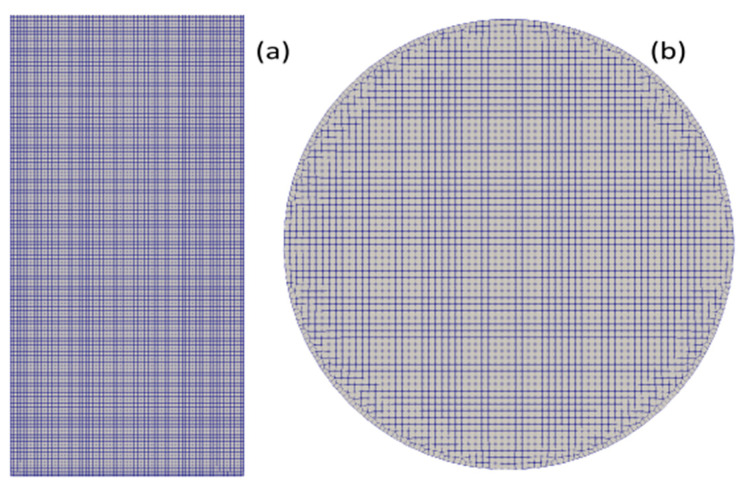
Computational grid of the column: (**a**,**b**) the longitudinal and the transversal plans, respectively.

**Figure 2:**
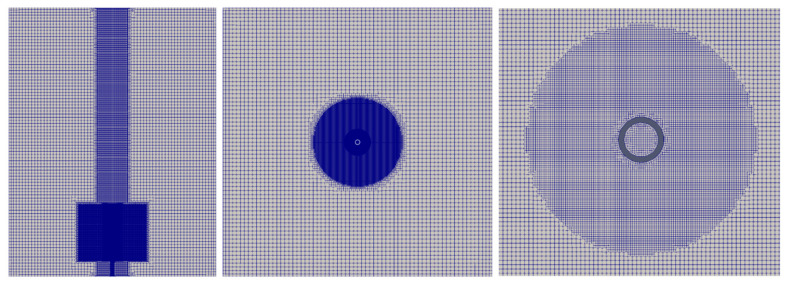
Computational grid of the single bubble injection domain.

**Figure 3:**
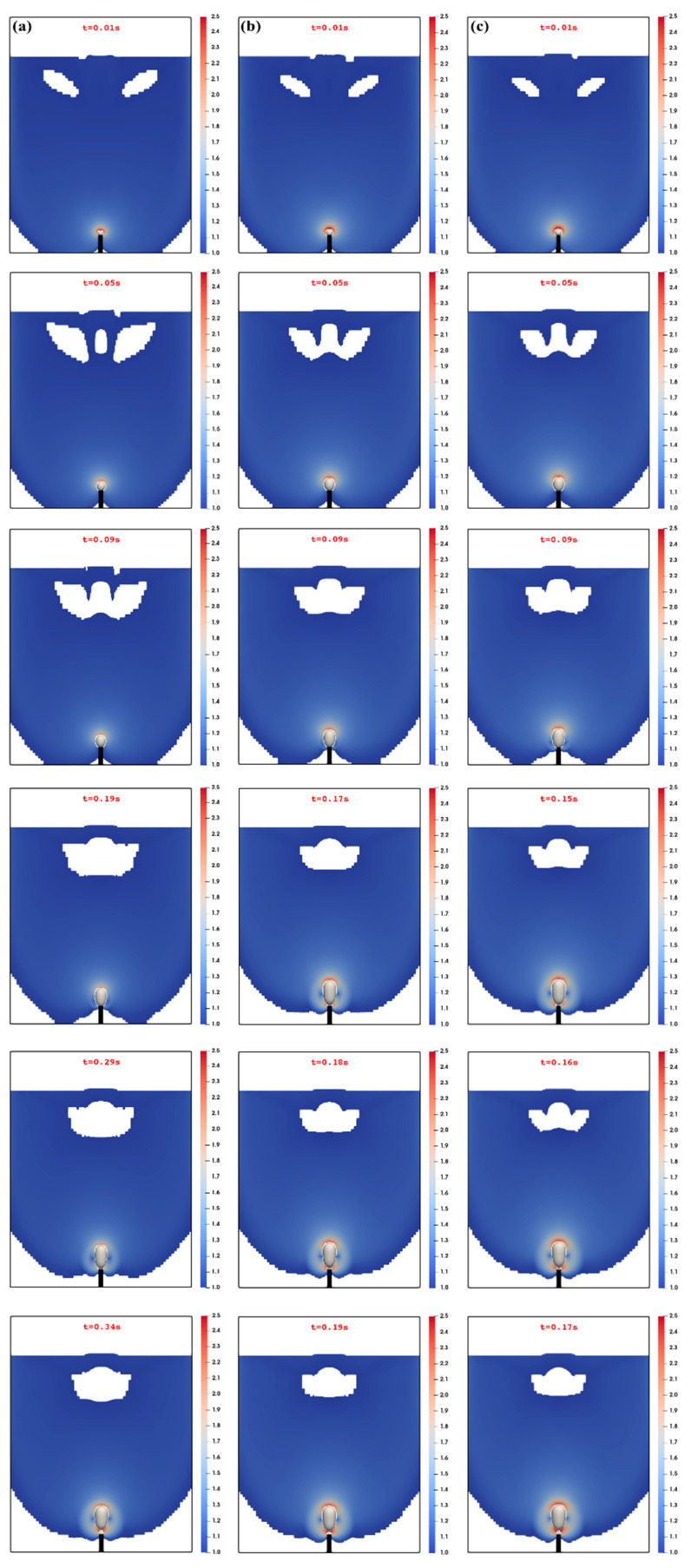
Sequences of the bubble formation in the aqueous solution of the xanthan gum with a dependence on the injected gas volumetric flow rate: (**a**) 7.89 × 10^−7^, (**b**) 1.60 × 10^−6^ m^3^/s, and (**c**) 1.98 × 10^−6^ m^3^/s. The threshold region represents the liquid region colored with the ratio of the second invariant of the viscous tensor to the yield stress of the xanthan gum (i.e., ${\left|{II}_{\tau_{1}}\right|}^{\frac{1}{2}}/\tau_{0}$).

The column is filled up with 0.68 kg of slurry, at an initial height of 0.17 m above the distributor plate. Summarized in Table 2 are the nine flow rates used in this work, which covers a broad range of typical gas influx rates in a wellbore (see, e.g., Bonett and Pafitis, 1996 [4]). Equations (26)–(37) are solved numerically on unstructured hexa-dominated grids (see Figure 1, Figure 2 and Figure 3) that are generated using the open-source code, cfMesh. Given in Table 3 is the summary of the mesh densities along with the grid spacings (distributed over the flow domain) that are used for the calculation. A variable time step with the maximum $\delta t=2.5\times 10^{-4}$ s is used to advance the solutions; this ensures that the Courant–Friedrichs–Lewy (CFL) remains below 0.2 and is allowed to resolve the fastest capillary waves in the domain (and subsequently to avoid undesirable oscillation phenomena of the interfaces caused by surface tension), as the time step remains smaller than the capillary time (see, e.g., Galusinski and Vigneaux, 2008 [39]; Brackbill et al., 1992 [30]).

Although Papanastasiou’s ϵ-dependent viscosity $\mu_{1,\epsilon }$ regularization technique is employed in this work, a parametric study is not carried out. ϵ is selected as small as $10^{-6}$ in order to limit plausible spurious effects on the bubble shapes (see, e.g., Dimakopoulos et al., 2013 [40]). The convective terms in the momentum equations, as well as the transport equation of the volume fraction, are discretized using the second order “linear” scheme. Spatial gradients are also discretized using the second order “linear” scheme (central differences with linear interpolation). The unsteady terms are discretized with a backward Euler scheme. Table 4 summarizes the descriptions of the quantities used throughout the manuscript.

## 5. Results

### 5.1. Formation of a Single Bubble and Its Growth

Prior to investigating the motion of bubbles in the cement slurry, the experimental work of Terasaka and Tsuge (2001) [18] on a single bubble formation and growth in a stagnant yield stress fluid is used to verify the results from the numerical simulations of the equations presented in the previous section. Terasaka and Tsuge (2001) [18] inject nitrogen gas through a cylindrical nozzle centered at the bottom of a square cross-section column, which is filled with an aqueous solution of xanthan gum; it is assumed that the fluid behaves as a yield stress fluid whose rheological data are fitted with the Herschel–Bulkley model. The column cross-section is 10 × 10 cm^2^, while the diameter of the nozzle is 1.5 mm. The properties of the fluid are summarized in Table 5. The depth of the aqueous solution above the top of the nozzle is 10 cm. Three different gas flow rates, namely 7.89 × 10^−7^, 1.60 × 10^−6^ m^3^/s, and 1.98 × 10^−6^ m^3^/s corresponding to gas orifice velocities of about 0.45, 0.91, and 1.12 m/s, respectively, are used, and the bubble volumes at the detachment are compared against the measurements from Terasaka and Tsuge [18].

The calculations are performed on an unstructured hexa-dominated computational mesh, with the smallest cells concentrated around the injection nozzle and along the axial region of the column (see Figure 2) to ensure an adequate resolution of the formation and growth of the bubble as the gas penetrates the fluid, causing strong velocity gradients. Thus, the cross-section of the gas injection nozzle is clustered with 16 cells such that the grid size in the injection nozzle and surroundings is ∆≃0.094 mm. The largest cells (i.e., with spacing ∆≃1.5 mm) are distributed within the outer region along the column. The generated grid consists of 6,707,927 cells. The numerical schemes and the regularization outlined above remain the same for this case study.

**Figure 4:**
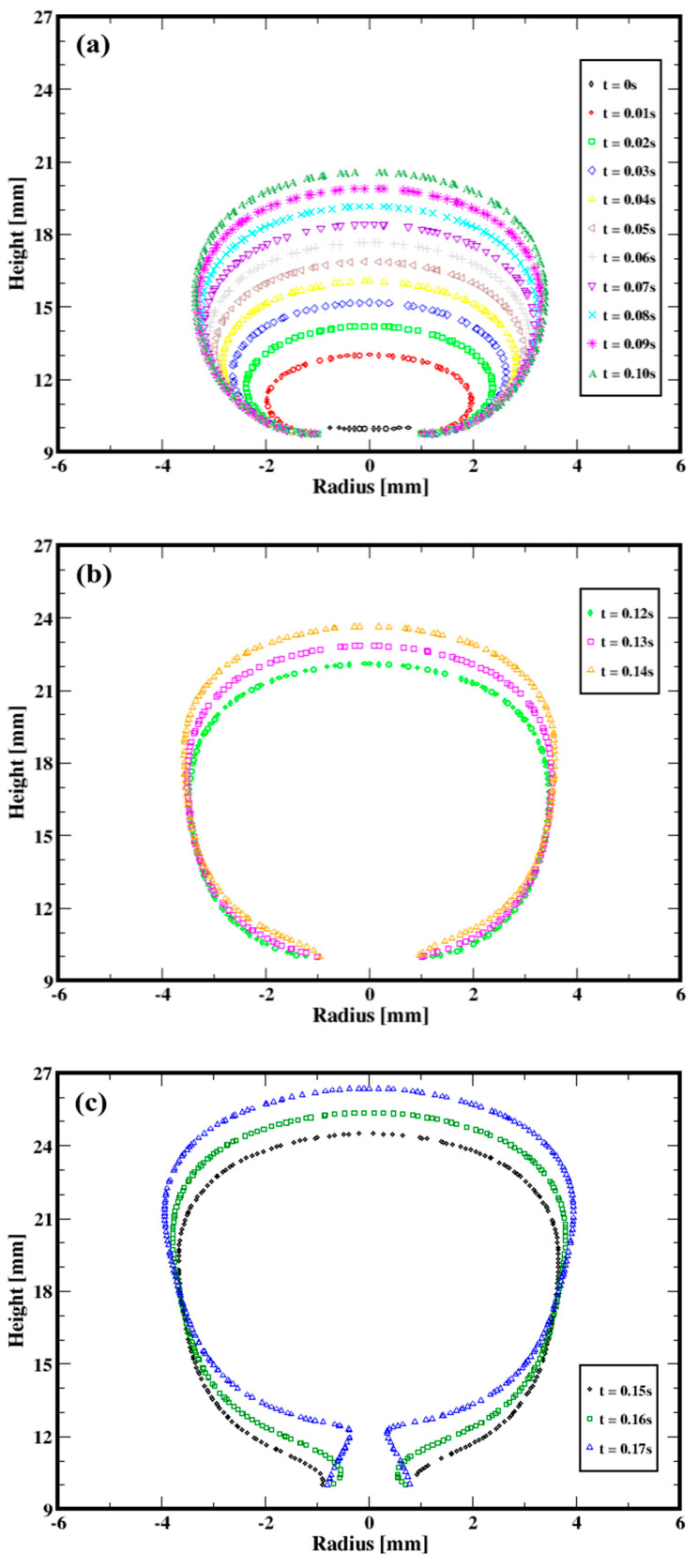
Sequential evolution of the interface between the injected gas and the aqeous solution of xanthan for the gas volumetric flow rate of 1.98 × 10^−6^ m^3^/s. (**a**) First stage (**b**) Second stage (**c**) Third stage.

The sequential snapshots of the bubble shown in Figure 3 exhibit markedly different stages during the bubble formation, which is consistent with the experimental observations (see, e.g., Terasaka and Tsuge, 2001 [18]), along with the flow region where the second invariant of the viscous tensor (i.e., ${\left|{II}_{\tau_{1}}\right|}^{\frac{1}{2}}$) exceeds the yield stress ($\tau_{0}$). Indeed, for each of these flow rates, the bubble appears first to increase in volume by expanding both radially and axially. Figure 4a, which illustrates the time evolution of the interface between the bubble and the fluid, further quantifies the growth of the bubble and the change in its aspect ratio (along with the range of shape). Along the second stage, which can be associated with the onset of a conical-like shaping of the bubble, the base diameter is progressively reduced (relative to the first stage) as a result of the upward stretching and elongations while the gas continues to be injected (see, e.g., Figure 4b for the quantitative change). Figure 4c shows that the third and the last stage of the formation of the bubble is essentially marked by the formation of the neck, which results in the detachment. 

**Figure 5:**
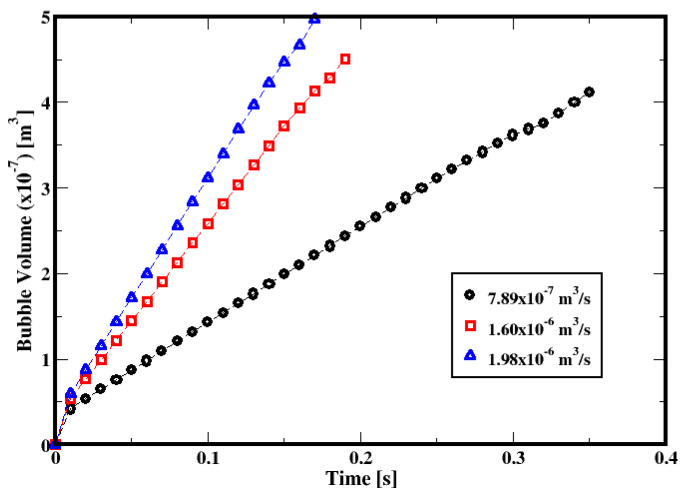
Dependence of bubble growth on the gas volumetric flow rate.

Figure 5 shows the bubble growth curves for the three flow rates. The growth rate increases with the gas injection rate, while the bubble formation period decreases.

**Figure 6:**
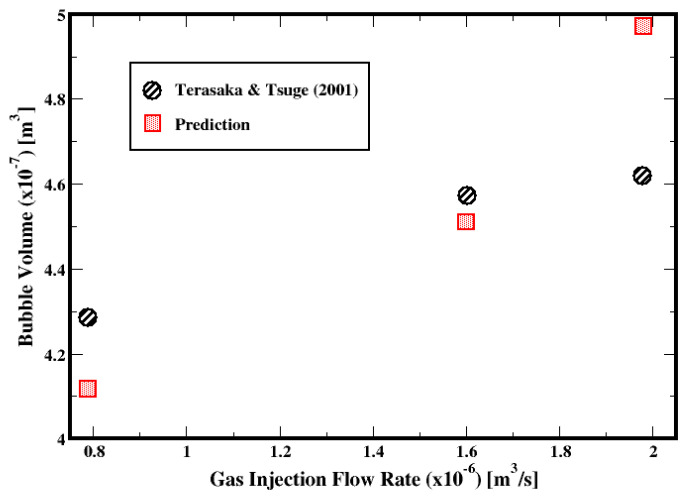
Prediction of bubble volume with respect to the gas volumetric flow rate [18].

Figure 6 compares the predicted volume of the bubbles at the time of the detachment against the measurements from Terasaka and Tsuge (2001) [18]. The deviations in the predictions associated with the flow rates 7.89 × 10^−7^, 1.60 × 10^−6^, and 1.98 × 10^−6^ m^3^/s are −3.93%, −1.34%, and 7.63%, respectively. Although the uncertainties in the measurements are not available, these deviations, which remain within ±10% margins relative to the measurements, can be regarded as relatively small considering that the mathematical models along with the numerical approach reasonably approximate the flow dynamics.

### 5.2. Multiple-Bubble Cement Slurry Column

#### 5.2.1. Mesh Refinement

The sensitivity of the solution on the grid resolution is based on the three grid densities summarized in Table 3. The generation of the finer grid (i.e., mesh #3), considered hereafter as the reference, is guided by the reasonable accuracy of the single bubble injection predictions. Indeed, the current “fine” mesh exhibits larger cells almost twice as small, while smaller cells are about twice as large, compared to single bubble injection. Given the considerable difference in the superficial velocity of the injected gas between the two systems (i.e., 2 to 4 order of magnitudes smaller with the cement column), the flow gradients can be assumed to be less strong within the column than for the single bubble formation and detachment. Therefore, the reference “fine” mesh is considered fine enough to capture the flow. The simulations are performed using the Herschel–Bulkley fluid model with a gas flow rate of 20 mL/min.

Table 6 compares the quantities of interest in this work, using these three grids. The extent of the yielded material, as well as the mean rise radial position and the rise angle, appears to be somewhat the same. The most significant deviations occur with the mean Sauter diameter and the mean rise velocity for which the deviations relative to the grid density #3 (i.e., the finer mesh) are quite large compared with the grid density #1 (i.e., the coarser mesh). In general, the deviations with the grid density #2 relative to the finer mesh are below 10%, which suggests that the details of the flow are adequately captured with the grid density #2.

#### 5.2.2. Flow Patterns

**Figure 7:**
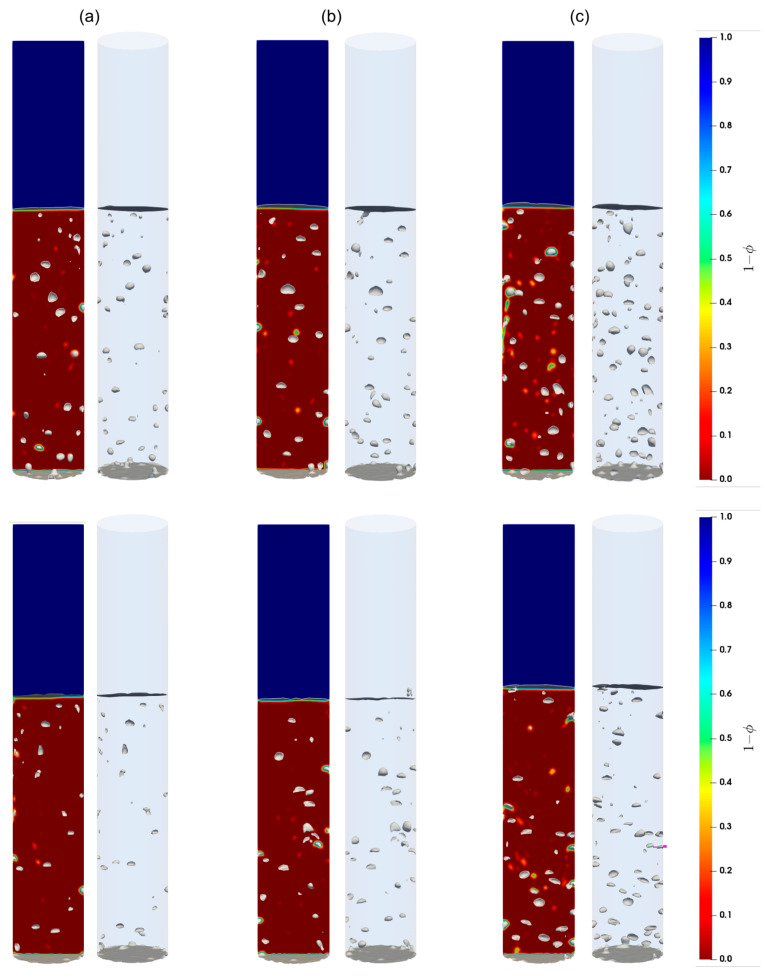
Contours of the cement slurry along with the air bubbles in the column; (**a**–**c**) correspond to the flow rates 2 mL/min, 4 mL/min, and 8 mL/min, respectively. Top and bottom parcels show the contours for the Herschel–Bulkley and the Bingham fluids, respectively.

**Figure 8:**
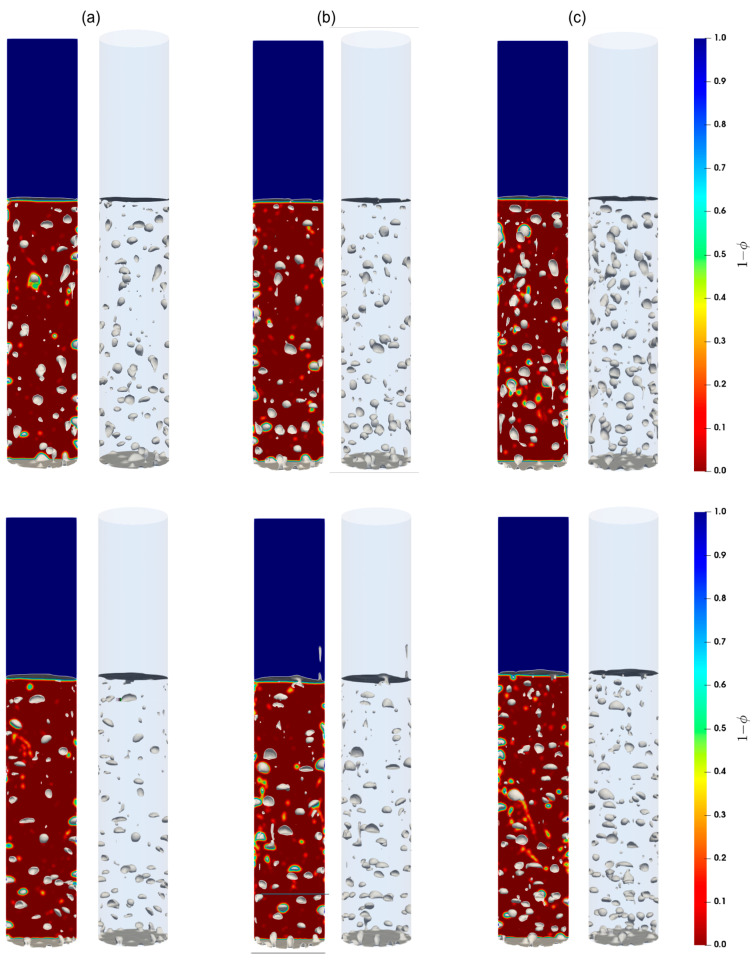
Contours of the cement slurry, along with air bubbles in the column; (**a**–**c**) correspond to the flow rates 15 mL/min, 20 mL/min, and 30 mL/min, respectively. Top and bottom parcels show the contours for the Herschel–Bulkley and the Bingham fluids, respectively.

**Figure 9:**
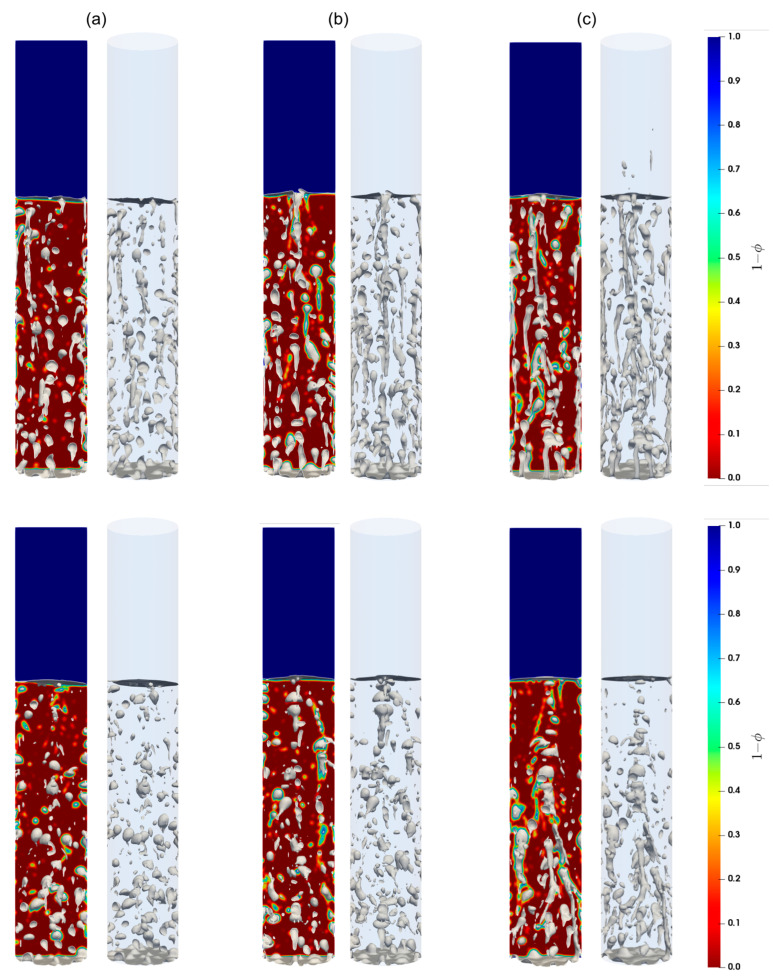
Contours of the cement slurry, along with air bubbles in the column; (**a**–**c**) correspond to the flow rates 60 mL/min, 90 mL/min, and 120 mL/min, respectively. Top and bottom parcels show the contours for the Herschel–Bulkley and the Bingham fluids, respectively.

Shown in Figure 7, Figure 8 and Figure 9 are the snapshots of the distribution of the volume fraction of the cement slurry and the bubbles in the cylindrical domain for different flow rates (see Table 2), along with the iso-surfaces (i.e., a three-dimensional surface passing through data of the same value) of 80% of the gas presence, which is considered at the external boundary of the gas bubbles. As we can see, different flow patterns can readily be identified for different lengths, sizes, and shapes of these iso-surface bubbles as the gas injection flow rate changes. Indeed, for low injection rates, relatively small bubbles rise toward the freeboard. With moderate flow rates, small to large bubbles appear to rise, while smaller bubbles often accompany the larger ones in their wakes. This is most likely a signature of the breakup of large bubbles as they rise. For high flow rates, the iso-surfaces exhibit stretched and elongated shapes toward the vertical axis as channeling paths for the gas; these bubbles appear to rise vertically in the column. Furthermore, these flow patterns remain consistent for both the Herschel–Bulkley and Bingham models used in this paper.

#### 5.2.3. Flow Behavior

It is known that an object moving through a yield stress fluid exhibits a surrounding envelope in which the material is yielded (see, e.g., Oldroyd, 1947 [41]; Beris et al., 1985 [42]; Piau, 2002 [43]; Tsamopoulos et al., 2008 [44]). In the context of a single freely rising bubble in a stagnant Herschel–Bulkley fluid, Dimakopoulos et al. (2013) [40] discuss the extent of the surrounding yielded region, as well as the unyielded areas around the bubble with respect to the Bingham number (for given Bond and Archimedes numbers), merging the circumstances of the unyielded material that may result in the entrapment of the bubble. Clearly, the evolution and the dynamics of the yielded (or unyielded) material can have strong implications on the dynamics of the rising bubbles. 

In this work, several bubbles rise in the column, and in order to quantify the fraction of the yielded material within a domain Ω, we introduce the conditional average of the material that yields within the domain:

(38) $$\langle \phi |{II}_{\tau_{1}}^{1/2}>\tau_{0}\rangle =\frac{\int_{\Omega }\phi H\left({II}_{\tau_{1}}^{1/2}-\tau_{0}\right)dV}{\int_{\Omega }\phi dV}$$

where H is the Heaviside function (i.e., equal to 1 when ${II}_{\tau_{1}}^{1/2}>\tau_{0}$ and is zero otherwise); ϕ is the volume fraction of the cement slurry. ${II}_{\tau_{1}}\left(=\frac{1}{2}\tau_{1}:\tau_{1}\right)$ is the second invariant of the stress tensor $\tau_{1},$ and $\tau_{0}$ is the yield stress of the material.

**Figure 10:**
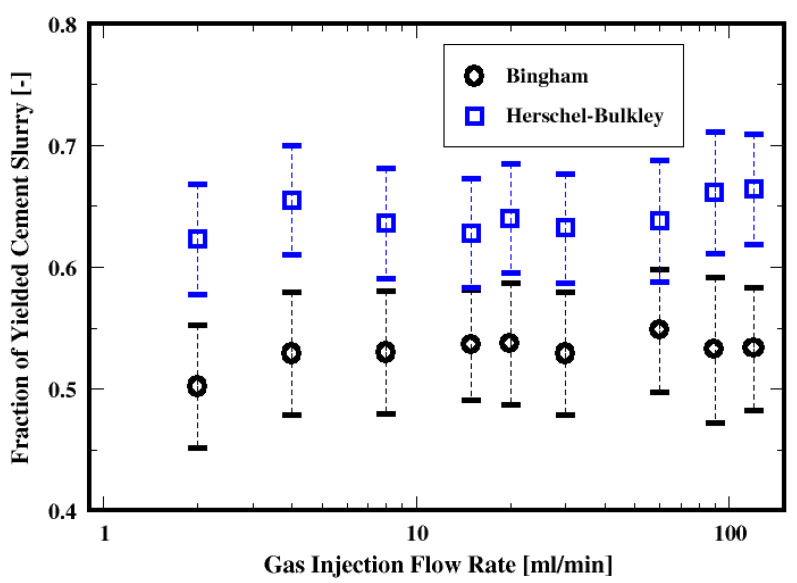
Dependence of the yielded material on the flow rate for the two fluid models.

Shown in Figure 10 is the dependence of the yielded cement slurry in the column for both the Bingham and the Herschel–Bulkley fluids. The increase in the gas flow rate seems to indicate that the fraction of the yielded material is virtually independent of the flow rates investigated here, since the differences appear marginal. However, it can be observed that approximately more than 10% of the material is yielded when using the Herschel–Bulkley model than the Bingham fluid. This difference in the yielded material may be related to the yield stress $\tau_{0}$ of the Bingham fluid (for this slurry), which is approximately twice for the Herschel–Bulkley model (see Table 1). Note that in this paper, we focus on the behavior of the bubble, its rise velocity and rise angle, etc. This figure also shows the change in the conditional average of the material that yields in the column with respect to the flow rate. Following the previous studies (also shown in the single bubble study in the current work), the rising of a bubble causes the material to locally yield. With the swarm of rising bubbles, the yielding behavior of the material would somewhat exhibit a dependence on the number of the bubbles, if one assumes an additive influence. However, it appears that such an effect (if any) is not shown, given that the flow rate increase results in marginal differences. In our next study, we will present detailed analysis of the flow field for the velocity, pressure fields, etc.

#### 5.2.4. Bubble Statistics

##### Size Distribution

**Figure 11:**
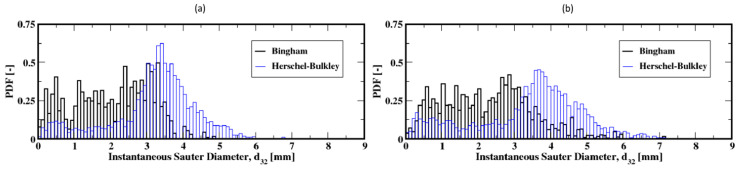
Distribution of the instantaneous Sauter diameter of the air bubbles in the column; (**a**,**b**) correspond to the flow rates 2 mL/min and 4 mL/min, respectively.

**Figure 12:**
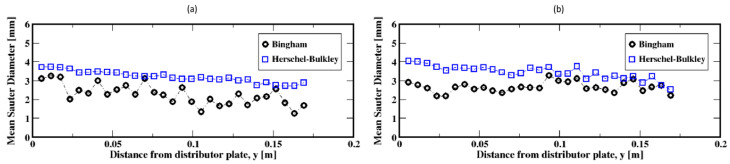
Mean Sauter diameter of the air bubbles in the column; (**a**,**b**) correspond to the flow rates 2 mL/min and 4 mL/min, respectively.

**Figure 13:**
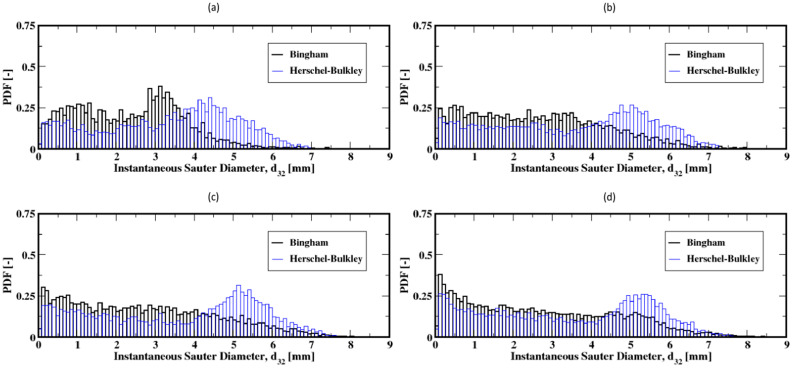
Distribution of the instantaneous Sauter diameter of the air bubbles in the column; (**a**–**d**) correspond to the flow rates 8 mL/min, 15 mL/min, 20 mL/min, and 30 mL/min, respectively.

The distribution of the air bubble Sauter diameters (i.e., volume-to-surface ratio equivalent diameter) are plotted in Figure 11, Figure 13 and Figure 15 for different gas flow rates. For low flow rates (identified here as 2 mL/min and 4 mL/min), the bubble size distributions mainly exhibit more than one mode (which is defined as a commonly found value of the distribution) for both the Herschel–Bulkley and the Bingham fluid models (see Figure 11). Both large and small size bubbles appear regardless of the fluid model, although a third mode is also apparent for the Bingham fluid. Furthermore, large bubbles are systematically larger for the Herschel–Bulkley model compared to Bingham. Figure 12a,b also show the average Sauter diameter along the column where the bubble sizes decrease as they rise toward the freeboard, regardless of the fluid model. 

**Figure 14:**
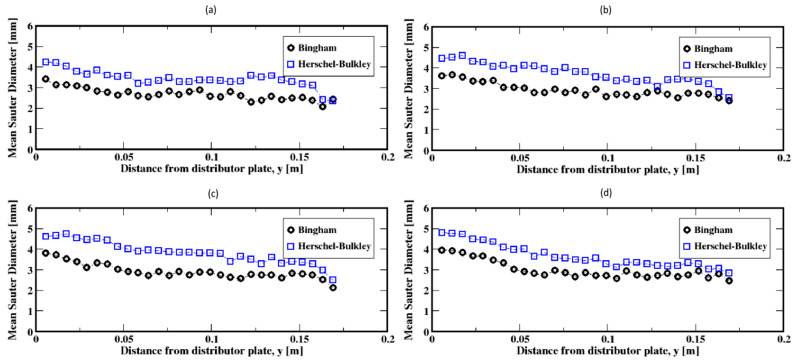
Mean Sauter diameter of the air bubbles in the column; (**a**–**d**) correspond to the flow rates 8 mL/min, 15 mL/min, 20 mL/min, and 30 mL/min, respectively.

The bubble size distributions shown in Figure 13a–d, associated with gas flow rates of 8, 15, 20, and 30 mL/min, exhibit an interesting bimodal trend of large and small bubble sizes marked with a progressive decrease in the frequency of large bubbles to the benefit of the small ones. This trend is independent of the fluid model used in this work. The apparent difference is the slightly larger bubbles for the Herschel–Bulkley model. Plotted in Figure 14a–d are the average Sauter diameters of the bubbles along the column. The exhibited unique pattern of decreasing bubble sizes with the distance above the distributor plate is indicative of a rising dynamics mostly controlled by breakup events in the column. 

**Figure 15:**
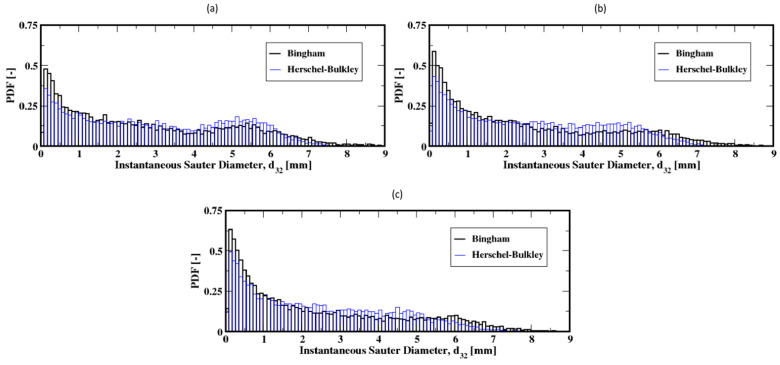
Distribution of the instantaneous Sauter diameter of the air bubbles int the column; (**a**–**c**) correspond to the flow rates 60 mL/min, 90 mL/min, and 120 mL/min, respectively.

**Figure 16:**
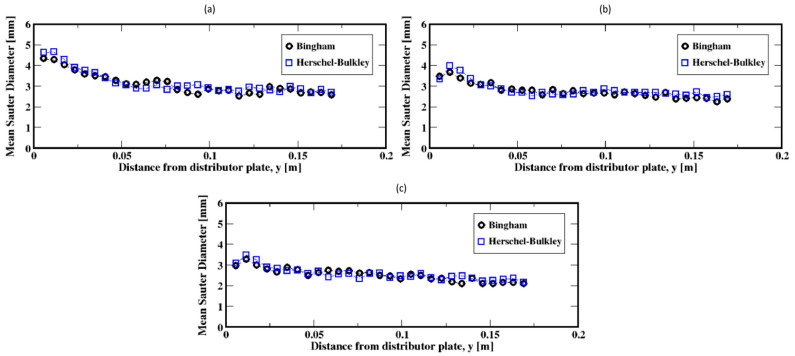
Mean Sauter diameter of the air bubbles in the column; (**a**–**c**) correspond to the flow rates 60 mL/min, 90 mL/min, and 120 mL/min, respectively.

Further increases in the gas flow rate (e.g., to 60, 90, and 120 mL/min) result in exponential-like size distributions of the Sauter diameters of the bubbles, both for the Herschel–Bulkley and the Bingham models (see Figure 15a–c). This indicates that small Sauter diameter bubbles populate the column, regardless of the fluid rheology. However, the slow decrease in the average size of the bubbles along the column, as shown in Figure 16a–c, suggests that the small Sauter size bubbles are distributed throughout the column following the breakup events of the bubbles as they rise toward the freeboard.

**Figure 17:**
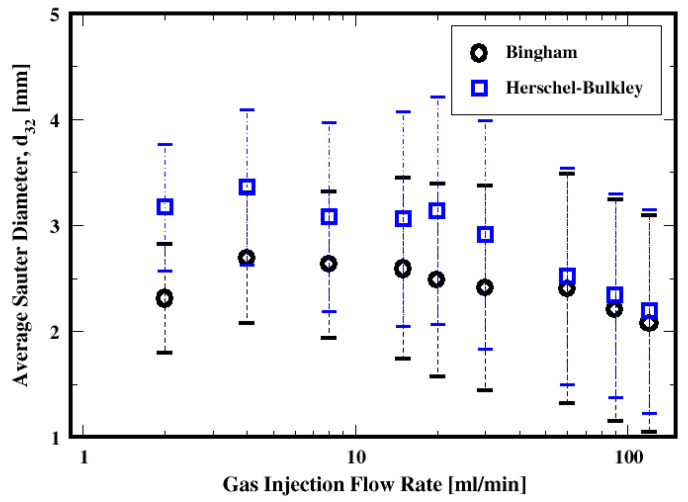
Dependence of the mean Sauter diameter on the flow rate and the fluid model.

Both the influence of gas flow rate and the cement properties on the average Sauter diameters of the bubbles are summarized in Figure 17. It is apparent that regardless of which model is used, the average bubble Sauter diameter decreases with the increase in the gas flow rate. Furthermore, relatively large dispersions are shown around these average diameters, thus indicating a broad range of bubbles evolving in the column. The difference in the fluid properties results in smaller average Sauter diameters for the Bingham fluid relative to the Herschel–Bulkley model. This is more pronounced for the moderate gas flow rates.

##### Aspect Ratio Distribution

The shapes of the rising bubbles are studied by looking at the distributions of the instantaneous bubble aspect ratio (i.e., ratio of the maximum horizontal length to the maximum vertical length) for different gas flow rates and fluid models. 

**Figure 18:**
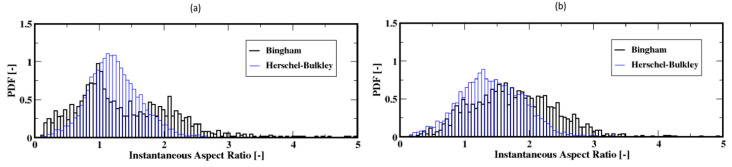
Distribution of the instantaneous aspect ratio of the air bubbles; (**a**,**b**) correspond to the flow rates 2 mL/min and 4 mL/min, respectively.

**Figure 19:**
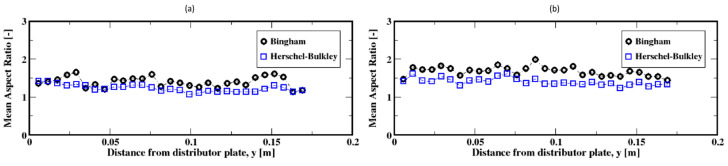
Mean aspect ratio of the air bubbles; (**a**,**b**) correspond to the flow rates 2 mL/min and 4 mL/min, respectively.

The distributions of the aspect ratios at low flow rates, which do not exhibit a unique shape, are plotted in Figure 18a,b. They show that the bubbles are most frequently wide/oblate and especially wider for the Bingham fluid compared to the Herschel–Bulkley fluid model. For these low flow rates, the average aspect ratio is presented in Figure 19a,b; they show that on average the bubbles tend to rise with moderately stable wide shapes for the Herschel–Bulkley fluid. Additionally, the bubbles appear to detach from the distributor plate with wide shapes regardless of the fluid model used. 

**Figure 20:**
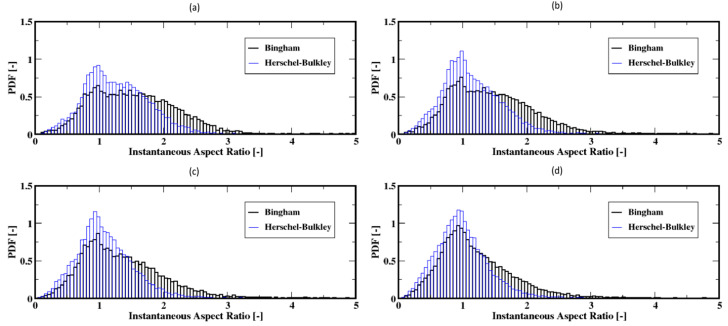
Distribution of the instantaneous aspect ratio of the air bubbles; (**a**–**d**) correspond to the flow rates 8 mL/min, 15 mL/min, 20 mL/min, and 30 mL/min, respectively.

**Figure 21:**
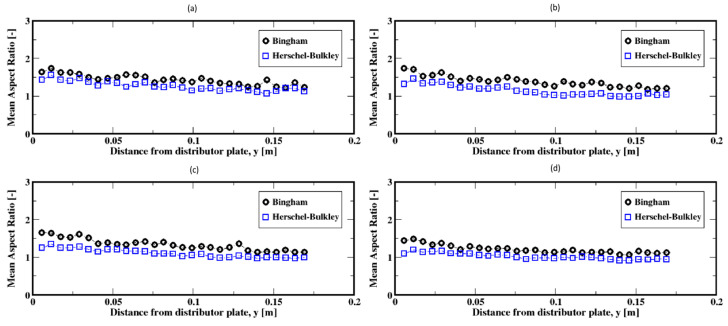
Mean aspect ratio of the air bubbles; (**a**–**d**) correspond to the flow rates 8 mL/min, 15 mL/min, 20 mL/min, and 30 mL/min, respectively.

As the gas flow rate increases, the aspect ratio distributions, Figure 20a–d, exhibit a shift toward the smaller ratios that peak around unity. This suggests that the bubbles are mostly of spherical shape and also that dependence of the shape of bubbles on the fluid model appears to fade out. Figure 21a–d present the average aspect ratios of the bubbles, which exhibit quite stable aspect ratios despite noticeable decreases. This indicates that the bubbles undergo smaller deformations (i.e., change in shape) after their detachment from the plate up to the freeboard. This trend is independent of the fluid model. However, the bubbles are wider for the Bingham fluid when compared to the case of the Herschel–Bulkley fluid for each of these moderate gas flow rates.

**Figure 22:**
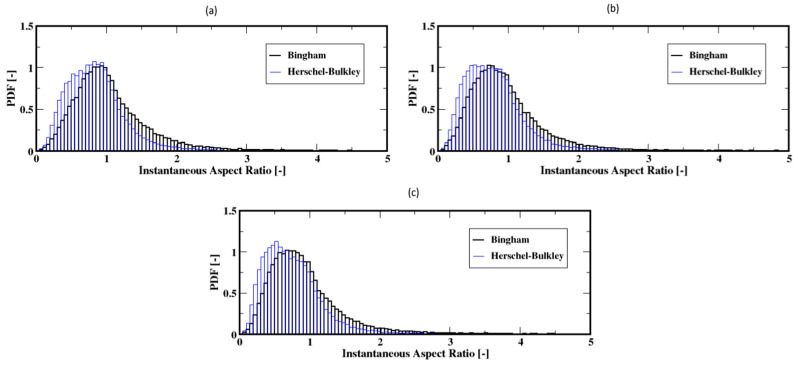
Distribution of the instantaneous aspect ratio of the air bubbles; (**a**–**c**) correspond to the flow rates 60 mL/min, 90 mL/min, and 120 mL/min, respectively.

**Figure 23:**
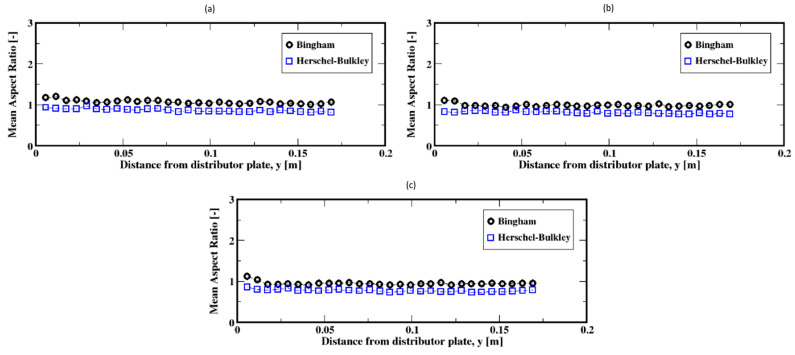
Mean aspect ratio of the air bubbles; (**a**–**c**) correspond to the flow rates 60 mL/min, 90 mL/min, and 120 mL/min, respectively.

For the higher flow rates, the distributions mostly shift toward the range of smaller ratios with most frequent values below the unity for the aspect ratio for both the Herschel–Bulkley and the Bingham fluids (see Figure 22a–c). That is, the bubbles are mostly elongated in the vertical direction, regardless of the fluid model used. Furthermore, the average aspect ratios along the column show that the bubbles tend to rise with stable elongated shapes but also that the Herschel–Bulkley fluid results in more elongated shapes compared to the Bingham fluid case (see Figure 23a,b).

**Figure 24:**
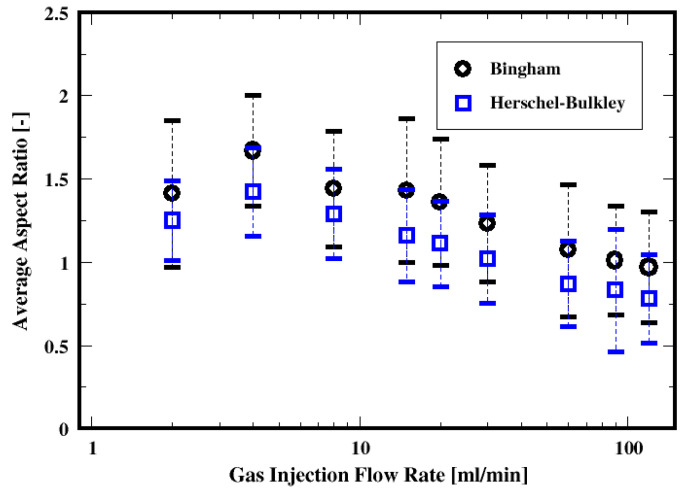
Dependence of the mean aspect ratio of the air bubbles on the flow rate and the fluid models.

Shown in Figure 24 is the dependence of the average aspect ratio on the gas flow rate, the fluid model, and the dispersion associated with each distribution. These average aspect ratios monotonically decrease with increasing gas flow rate, regardless of the fluid model. This indicates a change in the shapes from wide (or “kidney-like”) shapes to elongated (or “finger-like”) shapes with increasing gas flow rate.

##### Bubble Rise Velocity

**Figure 25:**
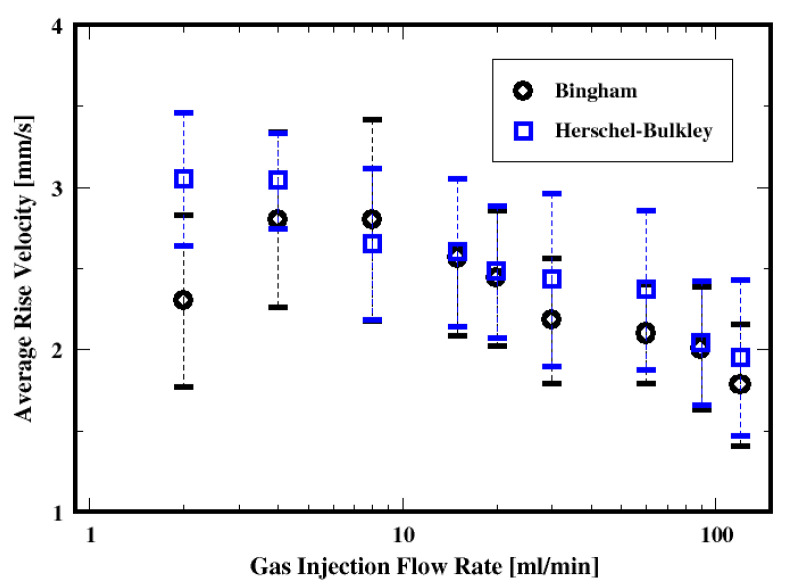
Dependence of the mean rise velocity of the air bubbles on the flow rate and the fluid model.

Figure 25 shows the dependence of the cross-sectional average bubble rise velocity on the gas flow rate. It is apparent that, on average, the rise velocities of the bubbles decrease with the gas flow rate, regardless of the fluid model. This is not surprising since the bubble size decreases as well with the gas flow rate (as shown in Figure 17), and the smaller bubbles rise more slowly than the larger ones. This dependence of the rise velocity on the bubble size (or volume) is well established and has been extensively discussed for a single bubble rising in non-Newtonian fluids (see, e.g., De Kee et al., 1990 [45]; Tsamopoulos et al., 2008 [44]; Amirnia et al., 2013 [46]; Ravisankar et al., 2022 [47]). Tsamopoulos et al. (2008) [44] discuss such an increase in the rise velocity with the bubble diameter; they attribute this to the decrease in the Bingham number. Moreover, they find that the rise velocity decreases with the Bingham number because of the increase in the drag coefficient.

**Figure 26:**
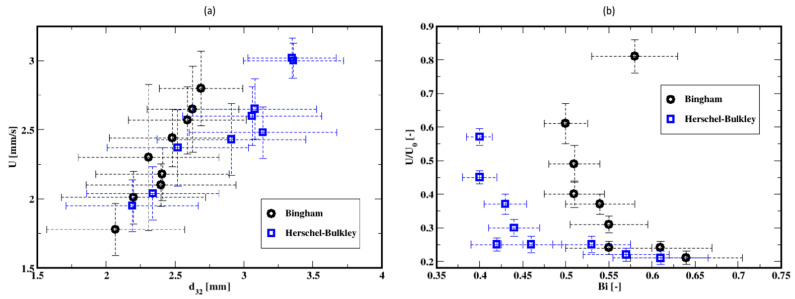
(**a**) Mean rise velocity of the air bubbles as a function of the mean Sauter diameter and (**b**) dimensionless mean rise velocity versus the Bingham number.

The mean rise velocity of the bubbles (which is defined as the arithmetic average of the bubble velocities calculated from the difference of consecutive axial centroid coordinates of the bubbles and divided by the time interval) is plotted as a function of the mean diameter in Figure 26a. Regardless of the fluid model used, the mean rise velocity increases with the mean diameter of the bubbles. However, the distributions of the rise velocities and the bubble diameters suggest that the description of the average rise dynamics must account for these dispersions and to a certain extent for additional mechanisms such as the breakup and coalescence of the bubbles. Buchholz et al. (1978) [48] report that bubbles in swarm have higher rising velocities than single bubbles (for a power-law Ostwald–de Waele fluid). With similar results, Yuan et al. (2021) [49] report that the group rising velocity increases with the number of bubbles, and also, the vertical chains become unstable for multiple bubbles because of the distinct oscillation of the uppermost ones. Vélez-Cordero et al. (2011) [50] discuss the dependence of the drag coefficient and the rise velocity on the interactions (e.g., drafting and kissing but not tumbling) between rising bubbles in shear-thinning fluid. The mean rise velocity plotted as a function of the mean bubble diameter (see Figure 26a) shows that the mean rising velocity is independent of the fluid properties for the smaller mean diameters, whereas the dependence becomes more apparent for larger mean diameters of the bubbles, where higher rise velocity occurs for the Bingham fluid. Similar mean rise velocity is noticed for small mean diameters for both the Herschel–Bulkley and the Bingham fluid descriptions of the cement slurry. This might have been controlled by the oscillating pressure at the inlet of the column (because of the zero gradient inlet pressure).

Shown in Figure 26b is the dependence of the dimensionless mean rise velocity (scaled by balancing the buoyancy and the viscous forces, i.e., $U_{0}=\rho gd_{32}^{2}/4\mu_{0}$ with $\mu_{0}=k{\left(U_{g,s}/d_{32}\right)}^{n-1},$ where $U_{g,s}$ is the gas superficial velocity) on the Bingham number (defined as $Bi=2\tau_{0}/\rho gd_{32}$). Following Tsamopoulos et al. (2008) [44], such a scaling aims at highlighting the influence of the bubble size. The Bingham number increases with the superficial gas velocity since the mean diameter of the bubbles decrease (see Figure 17), while the mean bubble rise velocity decreases. Thus, with the increase in the gas superficial velocity (or the gas volumetric flow rate), the dimensionless mean rise velocity of the bubbles decreases with the Bingham number, as shown in Figure 26b. Clearly, this is attributable to the increase in the drag as indicated in previous studies (see, e.g., Tabuteau et al., 2007 [51]; Tsamopoulos et al., 2008 [44]; Dimakopoulos et al., 2013 [40]; Mirzaagha et al., 2017 [52]).

##### Rise Angle

**Figure 27:**
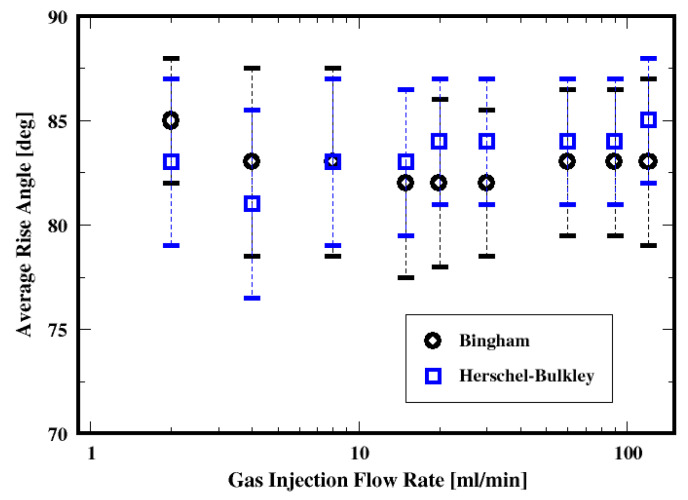
Dependence of the mean rise angle of the air bubbles on the flow rate and the two fluid models.

The average rise angle of the bubbles, which is defined as the arithmetic average of angles formed between the vectors of consecutive centroid coordinates of a generic bubble and the unit vector in the plane of the distributor plate, is plotted as a function of gas flow rate in Figure 27 for both the Herschel–Bulkley and Bingham fluids. The plots show that the bubbles rise throughout the slurry with average angles of approximately 81° to 84° relative to the distributor plate and with variances ranging between 6° and 9°. In other words, they mostly rise in almost vertical paths while exhibiting some oscillations (as suggested by the variances) toward the freeboard. Only the conditions of the low flow rates exhibit an average rising angle of approximately 66° with large variances of approximately 20° when using the Bingham model to describe the cement slurry. This suggests that the bubbles rise with more pronounced zigzagging trajectories. This behavior is consistent for these low flow rate conditions for the Bingham fluid, where the bubbles experience strong yield stress effects resulting in the breakup of the bubbles away from the distributor. Furthermore, it is worth noticing that instabilities in the trajectories of bubbles rising through non-Newtonian fluids are observed and discussed in the literature; they are thought to be associated with deformation and vortex shedding in the wake of the bubbles (see, e.g., Premlata et al., 2017 [53]; Sharaf et al., 2017 [54]). Premlata et al. [53] also report that, using quiescent shear-thinning liquids, increasing the shear-thinning behavior promotes path instabilities. In the present work, in addition to the shear-thinning behavior of the cement slurry, the gas injection introduces additional perturbations to the system that might interact with the vortex shedding in the wake of the bubbles.

##### Rising Position

**Figure 28:**
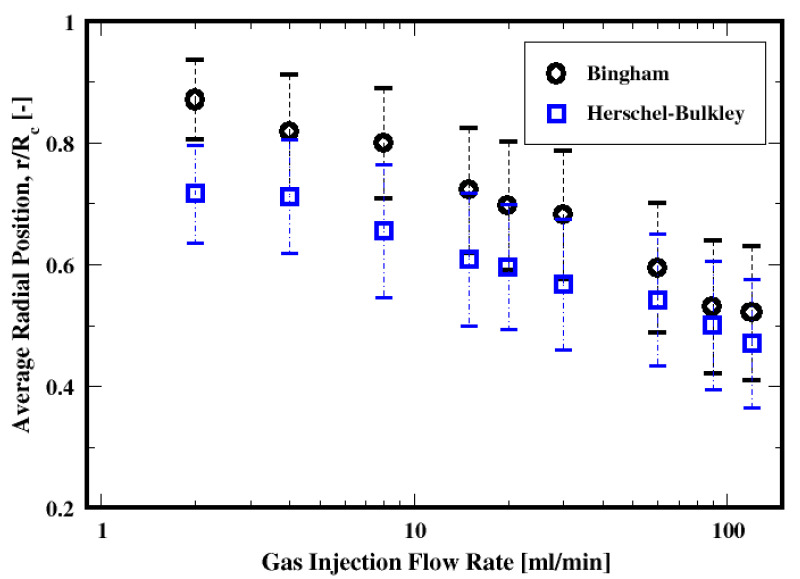
Dependence of the mean rise radial position of the air bubbles on the flow rate and the two fluid models. $R_{c}$ denotes the radius of the column.

Figure 28 shows the dependence of the average radial positions of the bubbles as they rise. First, this shows that the bubbles tend to rise throughout the peripherical regions of the column. Second, increasing the gas flow rate results in the broadening of the rising regions to the vicinity of the walls as illustrated by the monotonically decreasing of their positions toward the center of the column; this seems to be independent of the fluid models used here. 

Furthermore, for a given gas flow rate, the rising position behavior can be correlated to the resulting sizes of the bubbles. Indeed, it can be noticed that for a given flow rate, the larger the bubbles are, the farther the center of the bubbles are relative to the wall by comparison to the positions of the smaller ones. That is, when using the Herschel–Bulkley fluid, the bubbles are farther away from the wall, as opposed to the Bingham fluid case, since the bubbles are larger on average with the Herschel–Bulkley fluid (see Figure 17). 

#### 5.2.5. Influence of the Surface Tension

The influence of the surface tension on the bubbles’ geometrical characteristics and their rising dynamics are examined in the framework of the Herschel–Bulkley fluid model for gas flow rates in low and moderate ranges, namely 2 mL/min and 20 mL/min. We use three different values for the surface tension, σ = 0.025, 0.05, and 0.1 N/m, in addition to the previous value, σ = 0.07 N/m.

**Figure 29:**
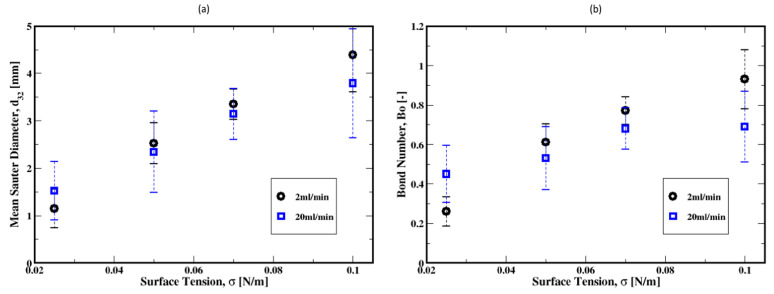
Dependence of the mean Sauter diameter of the air bubbles and the Bond number for gas flow rates of 2 mL/min and 20 mL/min. (**a**) the dependence of the bubble size on surface tension; (**b**) Dependence of the Bond number as a function of surface tension.

**Figure 30:**
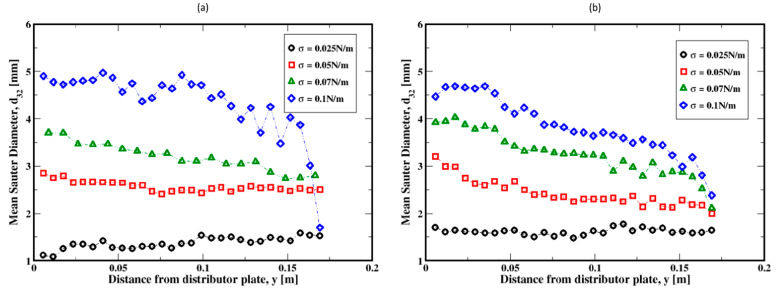
Dependence of the mean Sauter diameter of the air bubbles on the surface tension along the height of the column; (**a**,**b**) refer to the flow rates 2 mL/min and 20 mL/min, respectively.

Figure 29a illustrates the dependence of the bubble sizes on the surface tension, along with the Bond number defined as $Bo=\rho_{0}gd_{32}^{2}/4\sigma$ and plotted in Figure 29b. Figure 29a shows that the mean Sauter diameter of the bubbles monotonically increases with the surface tension. This is mainly due to the smaller bubbles detaching from the distributor plate as shown in Figure 30. In other words, the surface tension controls the initial sizes of the bubbles as they rise. The Bond number, plotted in Figure 29b as a function of the surface tension, remains on average below unity over the range of the surface tension investigated in this work. This suggests that the bubbles are mostly in regimes dominated by the surface tension.

**Figure 31:**
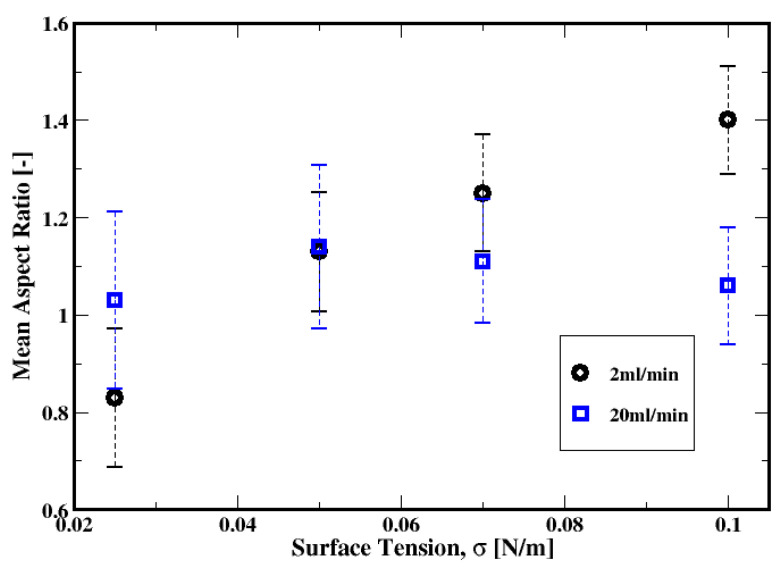
Dependence of the mean aspect ratio of the air bubbles on the surface tension for gas flow rates of 2 mL/min and 20 mL/min.

Meanwhile, the aspect ratio presented in Figure 31 shows that the increase in the surface tension results in substantial deformations of the bubbles at a low gas flow rate. Indeed, it is apparent for this low flow rate that the bubble shapes vary from an elongation-like shape in their vertical axes (more like a “finger”) to more oblate/flattened (or “kidney-like”) shapes from small to high surface tension. However, for the moderate gas flow rate, the dependence of the bubble aspect ratio on the surface tension appears weak and the bubbles remain quasi-spherical.

**Figure 32:**
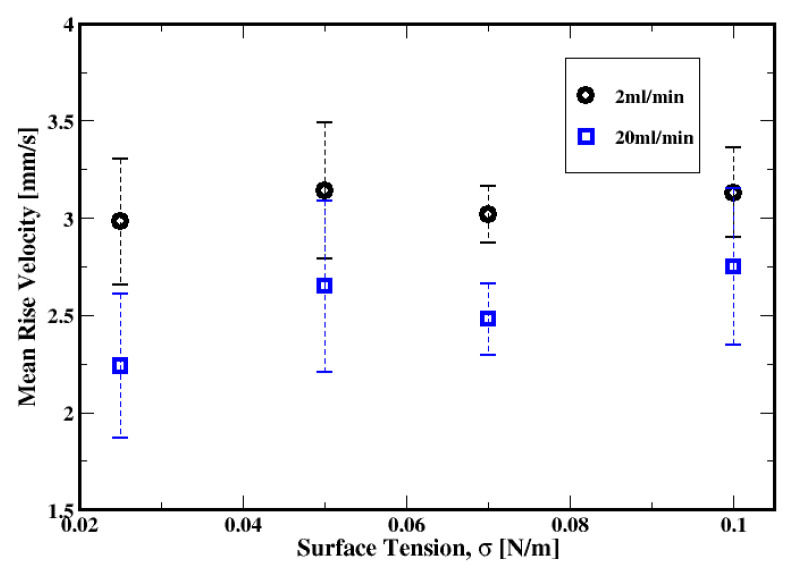
Dependence of the mean rise velocity of the air bubbles on the surface tension for gas flow rates of 2 mL/min and 20 mL/min.

The average rise velocity plotted in Figure 32 exhibits a weak dependence on the surface tension for the low and the moderate flow rates. The range of the Bond number (i.e., 0.2 to 1.1) used here for different surface tensions does not substantially change the rise velocity. Dimakopoulos et al. (2013) [40] report a non-monotonic change in the rise velocity of a single bubble rising in a stagnant Herschel–Bulkley fluid for Bond numbers ranging from 0.01 to 50. In our study, the weak dependence of the rise velocity on the surface tension may not only be related to the narrow range of Bond numbers used here but also a consequence of the inlet fluctuations of the pressure perturbating the slurry and the interactions between the swarm of the rising bubbles.

**Figure 33:**
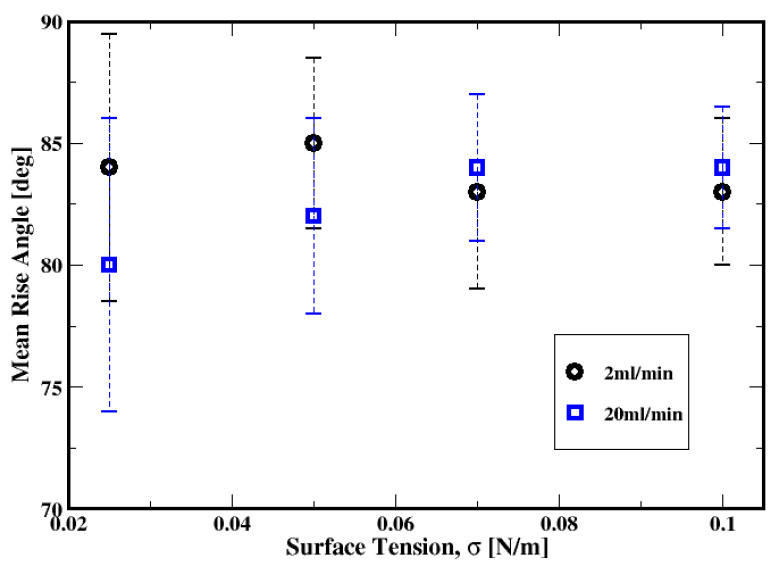
Dependence of the mean rise angle of the air bubbles on the surface tension for the gas flow rates of 2 mL/min and 20 mL/min.

Shown in Figure 33 is the mean rise angle, which also depicts a relatively weak dependence on the surface tension, especially at low flow rates. For the moderate flow rates, both the average and the extent of the variance of the rise angles indicate that zigzagging trajectories of the bubbles are more frequent with smaller values of the surface tensions.

## 6. Concluding Remarks

The motion of bubbles in a column of cement slurry in a freshly cemented wellbore is studied numerically. The two-phase system of the cement slurry and the air bubbles is described by using conservation equations of the mass and the linear momentum and solved in the framework of the volume-of-fluid (VOF) approach. Predictions of a single bubble formation and its growth in a yield stress fluid, namely, an aqueous solution of xanthan gum (Terasaka and Tsuge, 2001 [18]), shows good comparison with the experimental measurements of the bubble volume. The cement slurry is modeled as a Herschel–Bulkley fluid as well as a Bingham fluid for the sake of comparison. The simulations are performed over a wide range of gas flow rates, typical of gas influx rates in the wellbore.

The results show that increasing the gas flow rate results in mainly three identifiable flow patterns with varying lengths, sizes, and shapes of the air bubbles. Regardless of the fluid model, the mean bubble Sauter diameter decreases with increasing the gas flow rate. A unique pattern of decreasing bubble sizes at a distance above the distributor plate is noticed independently of the gas flow rate. The bubble shape changes from the wide-dominated case to vertically elongated shapes throughout the column when the gas flow rate increases. These results are similar for both the Herschel–Bulkley fluid and the Bingham fluid models.

The fraction of the yielded region appears virtually independent of the gas flow rate, but the extent of the yielded fluid is approximately 10% more with the Herschel–Bulkley model compared to the Bingham fluid. One plausible explanation of the non-dependence of the yielded fluid region on the gas flow rate could be due to the fluctuating nature of the pressure at the bottom of the column. The mean bubble rise velocities decrease with the gas flow rate, which can be attributed to the decrease in the bubble sizes with the gas flow rate, regardless of the fluid model. The dimensionless mean rise velocity decreases with the Bingham number, which is consistent with the results from previous works (see, e.g., Tabuteau et al., 2007 [51]; Dimakopoulos et al., 2013 [40]). The bubbles appear to rise more slowly in the Bingham fluid, probably because of the smaller bubble sizes or possibly because of the smaller extent of the yielded region found in the Bingham fluid.

Path instabilities of the bubbles, which are the deviations of the centroid motions of the bubbles from the vertical motion (i.e., zigzagging, or spiral paths), are found to be relatively weak and virtually independent of the gas flow rate. On average, the bubbles appear to rise with a relatively straight trajectory regardless of the fluid model used in this work. 

The influence of the surface tension shows that the surface tension dominates the buoyancy forces. However, the average rise velocity exhibits a weak dependence on the surface tension for the low and moderate flow rates.

Finally, the current predictions appear to match well with the experimental observations and exhibit the dependence of the bubbles motion on the gas flow rate. Furthermore, both the Herschel–Bulkley and Bingham models mostly exhibit similar trends. Moreover, a comparative analysis against the limiting case for Newtonian fluids warrants further investigations.

The influence of the microstructure, i.e., inhomogeneity of the cement slurry that is due to the cement particles in the suspension, expected to locally affect the yield stress, and the effective viscosity are planned for future work. 

## Figures and Tables

**Table 1:** Summary of the slurry properties for fitting the models (Headrick et al., 2023 [38]).

Model	$\tau_{0}$ [Pa]	$\eta_{p}$ [Pa·s]	k [Pa·s^n^]	n [-]
Herschel–Bulkley	12.87	-	1.96	0.69
Bingham	25.97	0.27	-	-

**Table 2:** Summary of the gas flow rate, along with the superficial gas velocity.

Gas Flow Rate (mL/min)	2	4	8	15	20	30	60	90	120
Superficial velocity (×10^−2^) (mm/s)	1.65	3.29	6.58	12.34	16.45	24.68	49.35	74.03	98.70

**Table 3:** Summary of the computational grids.

Mesh	Cell Count	${∆}_{min}$ [mm]	${∆}_{max}$ [mm]
1	338,424	0.32	1.31
2	650,840	0.24	1.05
3	1,517,008	0.17	0.79

**Table 4:** Summary of the descriptions of the quantities discussed throughout the manuscript. $V_{b}$ and $S_{b}$ are the volume and the surface of the bubble. $z_{b}^{\left(n\right)}$ denotes the vertical position of the bubble at a given time (nδt), δt being the time step. $U_{g,s}$ is the gas superficial velocity.

Quantity	Description
Bubble Sauter diameter, $d_{32}$	$d_{32}=6V_{b}/S_{b}$
Bubble aspect ratio, AR	$AR=\frac{max.horizontal\;length}{vertical\;max.length}$
Bubble rise velocity, $u_{b}$	$u_{b}=\frac{z_{b}^{\left(n+1\right)}-z_{b}^{\left(n\right)}}{\delta t}$
Scaling velocity, $U_{0}$	$U_{0}=\frac{\rho gd_{32}^{2}}{4\mu_{0}}$
Scaling viscosity, $\mu_{0}$	$\mu_{0}=k{\left(\frac{U_{g,s}}{d_{32}}\right)}^{n-1}$
Bingham number, Bi	$Bi=\frac{2\tau_{0}}{\rho gd_{32}}$
Bond number, Bo	$Bo=\frac{\rho gd_{32}^{2}}{4\sigma }$

**Table 5:** Rheological and physical properties of the xanthan gum aqueous solution.

$\mathrm{Density}\;(\rho_{1}$)	Surface Tension (σ)	$\mathrm{Yield}\;\mathrm{Stress}\;(\tau_{0}$)	Consistency (k)	Power-Law Exponent (n)	Temperature (T)
975 kg/m^3^	0.0551 N/m	10.2 Pa	4.06 Pa.s*^n^*	0.307	297

**Table 6:** Summary of mesh refinement. The deviations are calculated with respect to the finer mesh (i.e., mesh #3).

Mesh	#1		#2		#3	
	Mean	Deviation (%)	Mean	Deviation (%)	Mean	Deviation (%)
Yielded fraction (-)	0.61	−3.17	0.64	1.59	0.63	-
Sauter diameter (mm)	4.73	42.90	3.14	−5.14	3.31	-
Aspect ratio (-)	0.82	−18.81	1.11	9.90	1.01	-
Rise position, r/R (-)	0.51	−13.56	0.60	1.69	0.59	-
Rise velocity (mm/s)	3.65	31.77	2.50	−9.75	2.77	-
Rise angle (deg)	84	0	84	0	84	-

## Data Availability

Not applicable.

## Disclaimer

This project was funded by the U.S. Department of Energy, National Energy Technology Laboratory, in part, through a site support contract. Neither the United States Government nor any agency thereof, nor any of their employees, nor the support contractor, nor any of their employees, makes any warranty, express or implied, or assumes any legal liability or responsibility for the accuracy, completeness, or usefulness of any information, apparatus, product, or process disclosed, or represents that its use would not infringe privately owned rights. Reference herein to any specific commercial product, process, or service by trade name, trademark, manufacturer, or otherwise does not necessarily constitute or imply its endorsement, recommendation, or favoring by the United States Government or any agency thereof. The views and opinions of authors expressed herein do not necessarily state or reflect those of the United States Government or any agency thereof.

## Nomenclature

Symbol	Description
T	Cauchy stress tensor of the “one-fluid”
$A_{1}$	kinematical tensor
D	symmetric part of the velocity gradient tensor
I	identity tensor
$\tau_{1}$	cement slurry phase stress tensor
$\tau_{2}$	gas (air) phase stress tensor
$⟦\Sigma ⟧$	interfacial interaction between the two phases
${II}_{2D}$	second invariant of the tensor 2D
${II}_{\tau_{1}}$	second invariant of the stress tensor $\tau_{1}$
p	pressure of the “one-fluid”
u	velocity of the “one-fluid”
$u_{1}$	velocity of the cement slurry phase
$u_{2}$	velocity of the gas (air) phase
$u_{r}$	relative velocity between the two phases
n	unit normal vector
k	consistency of the cement slurry (for Herschel–Bulkley fluid)
n	power-law exponent of the cement slurry (for Herschel–Bulkley fluid)
ϕ	volume fraction of the cement slurry
σ	surface tension
κ	curvature of the interface between the two phases
δ	Dirac function
$\tau_{0}$	yield stress of the cement slurry
$\eta_{p}$	plastic viscosity of the cement slurry (for Bingham fluid)
μ	viscosity of the “one-fluid”
$\mu_{1}$	viscosity of the cement slurry phase
$\mu_{2}$	viscosity of the gas (air) phase
ρ	density of the “one-fluid”
$\rho_{1}$	density of the cement slurry phase
$\rho_{2}$	density of the gas (air) phase
ϵ	regularization parameter
δt	time step
∆	computational cell size
∇	gradient operator
$c_{\phi }$	compression factor
